# SOCIAL INTELLIGENCE TRAINING AND CUSTODIAL GRANDFAMILIES: A QUALITATIVE ANALYSIS OF RELATIONSHIPS

**DOI:** 10.1093/geroni/igad104.3069

**Published:** 2023-12-21

**Authors:** Megan Dolbin-MacNab, Gregory Smith, Frank Infurna, Britney Webster, Saul Castro, D Max Crowley, Carol Musil

**Affiliations:** Virignia Tech, Blacksburg, Virginia, United States; Kent State University, Kent, Ohio, United States; Arizona State University, Tempe, Arizona, United States; Diebold Nixdorf, Louisville, Ohio, United States; Arizona State University, Tempe, Arizona, United States; Pennsylvania State University, State College, Pennsylvania, United States; Case Western Reserve University, Cleveland, Ohio, United States

## Abstract

Custodial grandfamilies often experience relational challenges related to parenting and the grandparent-grandchild relationship that would benefit from intervention. Social intelligence training (SIT), which focuses on improving socio-emotional skills via online, self-guided modules, holds promise for helping grandfamilies address their relational challenges. The purpose of this qualitative study was to examine SIT skills that custodial grandmothers and grandchildren utilize in their interactions and the impact of SIT on their relationship. The sample consisted of 27 dyads of custodial grandmothers and adolescent (ages 11 to 18) grandchildren enrolled in an RCT examining the effectiveness of SIT. Dyads who completed the SIT were randomly selected and completed individual, open-ended interviews over the telephone, after post-test but prior to the 3-month follow-up. Results of a thematic analysis revealed several themes related to the SIT skills grandmothers and grandchildren were utilizing including taking and understanding the other’s perspective, attending to the nonverbal communication, engaging in active listening, reflecting on their treatment of each other, and slowing down communication to think before speaking. Analyses further revealed that grandmothers and grandchildren noted SIT-related improvements in their relationship, with themes reflecting improvements in the quality of communication, the frequency and intensity of conflict, understanding of each other, and sense of emotional connectedness. Those dyads that did not report relational improvements had a strong relationship prior to completing the SIT or the grandchild did not fully engage in the SIT. Directions for research and family-based interventions with grandfamilies will be discussed. [Funded by the National Institute on Aging (R01AG054571)]

